# A two-tiered targeted proteomics approach to identify pre-diagnostic biomarkers of colorectal cancer risk

**DOI:** 10.1038/s41598-021-83968-6

**Published:** 2021-03-04

**Authors:** Sophia Harlid, Justin Harbs, Robin Myte, Carl Brunius, Marc J. Gunter, Richard Palmqvist, Xijia Liu, Bethany Van Guelpen

**Affiliations:** 1 Department of Radiation Sciences, Oncology, Umeå University, 901 87 Umeå, Sweden; 2 Department of Biology and Biological Engineering, Chalmers University of Technology, Gothenburg, Sweden; 3 Chalmers Mass Spectrometry Infrastructure, Chalmers University of Technology, Gothenburg, Sweden; 4 Section of Nutrition and Metabolism, International Agency for Research On Cancer, World Health Organization, Lyon, France; 5 Department of Medical Biosciences, Pathology, Umeå University, Umeå, Sweden; 6 Department of Mathematics and Mathematical Statistics, Umeå University, Umeå, Sweden; 7 Wallenberg Centre for Molecular Medicine, Umeå University, Umeå, Sweden

**Keywords:** Cancer epidemiology, Cancer, Biomarkers, Predictive markers

## Abstract

Colorectal cancer prognosis is dependent on stage, and measures to improve early detection are urgently needed. Using prospectively collected plasma samples from the population-based Northern Sweden Health and Disease Study, we evaluated protein biomarkers in relation to colorectal cancer risk. Applying a two-tiered approach, we analyzed 160 proteins in matched sequential samples from 58 incident colorectal cancer case–control pairs. Twenty-one proteins selected from both this discovery phase and the literature were then analyzed in a validation set of 450 case–control pairs. Odds ratios were estimated by conditional logistic regression. LASSO regression and ROC analysis were used for multi-marker analyses. In the main validation analysis, no proteins retained statistical significance. However, exploratory subgroup analyses showed associations between FGF-21 and colon cancer risk (multivariable OR per 1 SD: 1.23 95% CI 1.03–1.47) as well as between PPY and rectal cancer risk (multivariable OR per 1 SD: 1.47 95% CI 1.12–1.92). Adding protein markers to basic risk predictive models increased performance modestly. Our results highlight the challenge of developing biomarkers that are effective in the asymptomatic, prediagnostic window of opportunity for early detection of colorectal cancer. Distinguishing between cancer subtypes may improve prediction accuracy. However, single biomarkers or small panels may not be sufficient for effective precision screening.

## Introduction

Colorectal cancer is one of the most common causes of cancer-related deaths in the world, and mortality is highly dependent on stage at diagnosis^1^. Early detection and treatment of colorectal cancer could therefore lead to decreased mortality rates world-wide. Many countries have implemented, or are in the process of implementing, age-based general screening programs, typically using colonoscopy, or fecal tests followed by endoscopy^2,3^. In addition to early detection, screening has major preventive and therapeutic effects, through the removal of precancerous and early malignant lesions. Improvements to general screening programs could, therefore, translate into substantial reductions in colorectal cancer incidence and mortality.

Currently, efforts to supplement colorectal cancer screening programs with blood tests for sub-clinical disease presence are ongoing^4,5^, including the FDA-approved test for Septin 9 DNA methylation^6^. Such tests use diagnostic biomarkers, i.e. biomarkers of disease, as an acceptable, minimally invasive and resource-effective means of selecting screening participants for colonoscopy. Another approach to refining general screening programs is through population-based risk stratification, in the hope of identifying higher-risk groups for earlier and/or more frequent screening. Risk algorithms using personal data such as age, family history of cancer, genetic risk variants and lifestyle-related factors show some promise for improving risk prediction^7,8^, but have not achieved sufficient accuracy to majorly impact general screening programs^9^.

Blood-based biomarkers for risk prediction represent an enticing avenue in the ongoing effort toward effective risk stratification and personalized colorectal cancer screening. However, given the focus on diagnostic biomarkers, the bulk of research in the field has used samples collected in a clinical setting^5^, from patients with existing colorectal cancer. Markers detected may, therefore, not be applicable in the pre-carcinogenic and early-carcinogenic phases particularly relevant for risk stratification. Some studies have used a screening setting, in which individuals with colorectal adenomas and polyps, not just carcinomas, are compared to those individuals free of neoplasms^10,11^, which may be a more promising approach. One venue that has shown some success is inflammation, a hallmark of cancer and an established etiological driver in colorectal cancer. A recent case-cohort study based in Japan, using a panel of 62 inflammatory biomarkers, identified a number of chemokines putatively related to subsequent colorectal cancer risk^12^. However, these have not been replicated in an independent sample.

In the present study, we used a two-tiered approach to colorectal cancer biomarker discovery and validation. Our primary aim was to identify novel biomarkers using large panels of inflammatory and cancer-related markers in a unique set of colorectal cancer cases and controls with time-matched, repeated, prediagnostic samples^13^, and to validate these in an independent sample from the same population. Our second aim was to validate findings reported in previous studies by incorporating them into a custom panel that also included our top findings.

## Materials and methods

### Study population

Participants were from the Västerbotten intervention programme (VIP)^14^ and the northern Swedish Monitoring of Trends and Determinants in Cardiovascular Disease (MONICA)^15^ cohort. The VIP is an ongoing preventive program (initiated in 1985) for cardiovascular disease and type-2 diabetes. All residents in Västerbotten County are invited for a primary health screening at 40, 50 and 60 years of age and at this time asked to fill out an extensive questionnaire covering lifestyle, diet and health as well as donate a blood sample for research purposes. The North Sweden MONICA project is part of the WHO MONICA and consists of cross-sectional questionnaire surveys and blood sample collections conducted in 1986, 1990, 1994, 1999, 2004, 2009 and 2014 (with a new collection planned for 2021). MONICA participants are randomly selected from the inhabitants of Västerbotten and Norrbotten in Northern Sweden.

Blood samples for both VIP and MONICA are collected in EDTA and Heparin tubes and aliquots of plasma, buffy coat and erythrocytes are frozen within 1 h of collection. Samples are stored at − 80 °C at the Northern Sweden Biobank (Biobanken Norr) in Umeå, Sweden. All samples are collected in the morning and participants of both cohorts are asked to fast for at least 8 h prior to sampling. If for some reason the participant has not fasted, or fasted for a shorter time-period, this information is noted in the accompanying sample file. Both VIP and MONICA are part of the Northern Sweden Health and Disease Study (NSHDS)^16^.

The study was approved by the regional ethical review board at Umeå University, Umeå, Sweden (Ref number: 2015/172-32 and 2015/391-32M). All study subjects provided written informed consent at recruitment, and the study was conducted in accordance with the Declaration of Helsinki.

### Study design

The discovery set included only VIP participants, and all cases were selected based on the following strict criteria: A primary colorectal cancer diagnosis within 5 years after the most recent sampling (excluding the last 3 months before diagnosis), at least two available blood samples in the biobank collected at least 10 years apart, and no other primary cancer diagnosis, except non-melanoma skin cancer, at the final date of follow-up (Dec 31st 2014). Controls were matched to cases based on age (± 12 months), sex and sampling dates (+/− 12 months for both sampling occasions). Controls had to be cancer free for at least 5 years after the colorectal cancer diagnosis of their index case or at the end of follow up, whichever came first. Only samples collected after at least 8 h of fasting were included, and no samples had been thawed prior to aliquoting for analysis. The original discovery sample set included 69 matched case–control pairs, all with time-matched repeated samples, and has been previously described^13^. Nine individuals failed in the proteomics quality control and were excluded together with their matched cases or controls. Thus, the final study population in the discovery phase consisted of 120 participants, 60 cases and 60 controls, all with time-matched repeated, pre-diagnostic samples. Later DNA methylation array analyses in another study using the same participants^17^ revealed identity mismatch between repeated samples of two individuals (one case and one control). Subsequent error analysis determined that the identity mismatch had occurred at the biobank, prior to sample shipment. Although the validation phase was already underway at that point, we reran the statistical analyses on the discovery set, excluding these case sets, for comparison.

The validation sample set was selected from a larger nested colorectal cancer case–control study, comprising 1010 case–control pairs (matched on age at and year of sampling, sex, study cohort, freeze thaw cycles and fasting status) from the VIP and MONICA cohorts, described in detail elsewhere^18^.

We selected 1000 samples from this nested case–control study, prioritizing sample plates to minimize the number of cases with peridiagnostic blood samples available in the related clinical colorectal cancer cohort, U-CAN^19^. This was done to reserve patients with both prediagnostic NSHDS and peridiagnostic U-CAN samples for possible future validation of potential novel diagnostic biomarkers. The final validation sample sent for analysis included 461 matched colorectal cancer case–control pairs, of which 39 had time-matched repeated samples.

### Outcome variables and covariates

Outcome data in both the discovery and validation phases were obtained by linkage to Swedish national registers (the Swedish Cancer Register, The Swedish Cause of Death Register and the Swedish Register of the Total Population). Colorectal cancer cases were identified using ICD-10 codes (18.0 and 18.2–18.9 for colon cancer, 19.9 and 20.9 for rectal cancer) and verified by a gastrointestinal pathologist (Richard Palmqvist). Data on disease stage and anatomical location were retrieved from the Swedish Colorectal Cancer Register and, in cases of missing data, from patient records. Molecular tumor data (*BRAF* V600E and *KRAS* mutations) were generated in house, as previously described^18^. Additional covariates were considered based on previously established associations with colorectal cancer risk and data availability. They included: age at sampling, sex, body mass index (BMI, based on height and weight measured by a health professional), self-reported smoking status, education, alcohol intake and physical activity.

### Sample collection and laboratory analysis

Blood samples were collected in EDTA tubes, centrifuged, aliquoted and frozen within 1 h of sampling. In the discovery phase of the study, plasma samples were analyzed for two panels of biomarkers using predesigned Proseek Multiplex immunoassays (Inflammation and Oncology II, Olink Proteomics, Uppsala, Sweden), as previously described^13^. We selected the Inflammation panel based on the role of inflammation in colorectal cancer etiology and progression, leading to the hypothesis that inflammatory biomarkers may have merit as potential risk predictive and/or early diagnostic biomarkers of colorectal cancer. The Oncology II panel was added to capture additional markers associated with cancer and cancer development but not included in the Inflammation panel. All proteins included in the commercially available Olink panels, including Oncology II and Inflammation, are pre-selected by Olink, and we thus had no influence on panel content. It is also worth noting that since our initial selection (performed in 2016), the number of available panels has increased substantially. The multiplex panels rely on Proximity Extension Assay (PEA) technology, which maintains specificity despite high multiplexing levels^20^.

All sample processing and quality control was performed by Olink Proteomics. Data were delivered as Normalized Protein eXpression (NPX) values on a log2 scale and pre-processed as described in previous publications^13,21^. Information about limits of detection (LOD) can be found online (http://www.olink.com). The full list of markers included in both panels, together with the percentage of samples that fell below the LOD, can be found in Supplementary Table S1.

For the validation phase of the study, we selected 21 biomarkers to be analyzed on a custom-made panel, designed for us by Olink proteomics (Supplementary Table S2). For the panel, we prioritized proteins from our own discovery phase that had an FDR of less than 0.25. Fifteen proteins passed this threshold and of these; three failed quality control in the multiplex design (TNFSF13, S100A4 and CEA). TNFSF13 and S100A4 were hence excluded from the validation phase. However, as CEA is a known, and used, colorectal cancer tumor marker we deemed it to be of strong interest and therefore decided to include it, but run the analysis as a single assay. Protein biomarkers selected from the literature filled the additional eight available spots on the custom panel, however there were several restrictions limiting our selection of markers from the literature. First, the markers had to be available on one of the pre-existing Olink panels, second, they had to pass quality control for multiplexing and third their concentration in plasma had to be in the right range as to not need dilution. The final selection of literature markers included eight proteins that fit these criteria and were selected from two previous publications^22,23^.

Assay performance for the custom-panel was assessed by Olink proteomics during the design stage and continuously during sample runs. Briefly, three internal controls were added to each multiplex plate and two internal controls to each singleplex plate in order to monitor the quality of assay performance and the quality of individual samples. The standard deviation from the internal controls was evaluated for each sample plate; if the deviation was above 0.2 NPX values, the plate was rerun. Individual samples were evaluated by determining the deviation from the median value of the controls, if this exceeded 0.3 NPX values the sample was excluded from the analysis. Controls in triplicate were used for calculations of Inter- and Intra-Assay Coefficients of Variability (CV). In order to reduce the impact of batch effects to a minimum, cases and their matched controls were always placed on the same plate. After quality control and pre-processing, data were delivered as NPX values. All included proteins are presented in Supplementary Table S2.

### Data pre-processing

In the discovery dataset all individuals contributed two samples each. For those individuals lacking covariate data at one of the sampling occasions, information from the other occasion was used to complete the dataset. In the validation dataset, missing data were observed for BMI (N = 9), smoking status (N = 21), level of education (N = 13), alcohol consumption (N = 116) and physical activity (N = 116). As most participants in the validation phase lacked repeated measures, we used multivariate imputation by chained equation (mice R-package), to replace the missing data, under the assumption that data were missing at random^18^. For continuous variables (BMI) we used predictive mean matching, whereas for multi-categorical (N > 2) variables (smoking status and level of education) Bayesian polytomous regression was used. Predictors originally included age, sex, sampling year, smoking status, BMI, education level, alcohol consumption and cohort (MONICA or VIP). To assess the robustness of our imputation method, we repeated the imputation step multiple times with different random starting samples and compared the results to analyses in which observations with missing data were excluded.

To reduce the influence of extreme outliers, relative protein concentrations were winsorized to the 1st and 99th percentile. To simplify comparisons between protein associations, the log2 NPX values were scaled to mean 0 and SD 1 prior to data analysis. Proteins with > 50% of values below LOD were removed from the dataset (Supplementary Table S1). For the remaining proteins, values below LOD were replaced by protein specific LODs (discovery data set) or individually reported and included in the analyses (validation data set).

### Statistical analysis

For each protein, conditional logistic regression was used to calculate an odds ratio (OR) of colorectal cancer risk. Multivariable models included smoking status, BMI, and education level as covariates. In the discovery phase, availability of repeated measurements for all participants allowed us to perform the analyses at two different time points. In the validation phase baseline values were used for all downstream analyses, unless otherwise specified. Pre-defined subgroup analyses were performed based on tumor location (colon/rectum), stage (I–II/III–IV), time to diagnosis (> 5 years/≤ 5 years after sampling) and molecular subtypes based on *KRAS* and *BRAF* mutations.

On the validation phase dataset, we applied logistic regression with Least absolute shrinkage and selection operator, LASSO, to identify a subset of informative features from 21 proteins. The penalty parameter was chosen using tenfold cross-validation with respect to predictive performance (area under the curve, AUC). Cross-validation was repeated 100 times with different training/test set partitions to accommodate for randomness in the partitioning. The lasso model was adjusted for age, sex, BMI, smoking status, and level of education which were therefore excluded from penalization. In order to evaluate our extended model, including covariates and the additional proteins selected by LASSO, we compared it to a model containing only the risk factors included as covariates in the logistic regression (age, sex, BMI, smoking status, level of education) and performed a likelihood ratio test. We also compared our models by plotting receiver operator curves (ROCs) and calculating the AUCs.

For the individuals in the validation set that had repeated measures we also conducted a longitudinal analysis using linear mixed models for the top proteins associated with colorectal cancer. We included subject ID and case–control pair ID as random effects, and BMI, smoking status, level of education, and time to diagnosis as fixed effects parameters. An interaction term between case–control status and time until diagnosis was included to investigate changes in protein levels over time between cases and controls. Time until diagnosis was defined as time of sampling until time at diagnosis, for cases, and as time from sampling until time of diagnosis for their matched case, for controls. Models were fitted using the lme4 R-package and the degrees-of-freedom and p-values were estimated by Satterthwaite approximation.

All p-values in the discovery phase were adjusted for multiple comparisons using the false discovery rate (FDR), q-value framework^24^. In the discovery phase of this study, q-values below 0.25 were considered for selection to the validation panel. In the validation phase, *p* values < 0.05 were considered statistically significant.

All computations were conducted in R v.3.6.0 (R Foundation for Statistical Computing, Vienna, Austria).

## Results

### Participant characteristics

Participant characteristics are shown in Table 1. Overall, there were no clear differences, in terms of baseline characteristics, between cases and controls. In the validation set, cases tended to have a higher BMI and lower education compared with controls. Neither smoking nor alcohol intake varied between cases and controls in either of the datasets. Age at baseline was approximately 10 years higher in the validation set (59.8 years) compared to the discovery set (49.9 years), due to the requirement of repeated samples (collected at 10 year intervals) in the discovery set. Also due to the study design, the mean age at colorectal cancer diagnosis was lower in the discovery set (60.6 years compared to 66.6 years in the validation set) (Table 1).

**Table 1:** Participant characteristics.

Variable	Discovery set (n = 116^a^)			Validation set (n = 900^a^)		
	Cases (n = 58)	Controls (n = 58)	*p*^b^	Cases (n = 450)	Controls (n = 450)	*p*^b^
**Cohort n (%)**			1.0			1.0
VIP	58 (100)	58 (100)		417 (92.7)	417 (92.7)	
MONICA	0 (0)	0 (0)		33 (7.3)	33 (7.3)	
**Repeated samples n (%)**						
Yes	58 (100)	58 (100)	1.0	38 (50)	38 (50)	
**Age median (range)**						
Baseline (years)	49.9 (39.5–52.5)	49.9 (39.7–52.4)	1.0	59.8 (29.7–74.5)	59.8 (29.8–74.9)	1.0
Follow up (years)	59.9 (49.9–60.5)	59.9 (49.9–60.6)	1.0	59.9 (39.9–73.0)^c^	60.0 (40.0–73.9)^c^	1.0
Diagnosis (years)	60.6 (50.2–65.1)	N/A		66.6 (40.4–89.6)	N/A	
**Sex n (%)**						
Men	32 (55.2)	32 (55.2)	1.0	240 (53.3)	240 (53.3)	1.0
**Anthropometrics median (range)**						
Height (cm)	172.0 (157.0–195.0)	170.5 (157.0–191.0)	0.9	171.0 (150.0–201.0)	170.8 (150.0–194.0)	0.6
Weight (kg)	75.50 (51.0–123.0)	75.85 (52.0–128.0)	0.9	77.00 (48.0–143.0)	75.20 (45.0–132.0)	0.1
**Body mass index median (range)**						
BMI (kg/m^2^)	25.32 (19.6- 37.8)	24.43 (18.8–41.3)	0.9	26.03 (17.78–43.04)	25.72 (17.15–44.62)	0.1
**Body mass index groups n (%)**						
< 18.5	0 (0)	0 (0)	0.3	2 (0.5)	9 (2.0)	0.2
18.5–24.9	27 (46.6)	32 (55.2)		168 (37.3)	170 (37.8)	
25–29.9	26 (44.8)	18 (31.0)		195 (43.3)	197 (43.8)	
≥ 30	5 (8.6)	8 (13.8)		80 (17.8)	70 (15.6)	
Missing^d^	0 (0)	0 (0)		5 (1.1)	4 (1.0)	
**Smoking n (%)**						
Current smoker	20 (34.5)	17 (29.3)	0.7	111 (24.7)	96 (21.4)	0.2
Former smoker	13 (22.4)	17 (29.3)		155 (34.4)	143 (31.8)	
Never smoker	25 (43.1)	23 (39.7)		174 (38.7)	201 (44.6)	
Missing^d^	0 (0.0)	1 (1.7)		10 (2.2)	10 (2.2)	
**Education n (%)**						
Elementary	26 (44.8)	26 (44.8)	0.8	332 (73.8)	307 (68.2)	0.1
Secondary	16 (27.6)	18 (31.1)		56 (12.4)	62 (13.8)	
Post-secondary	16 (27.6)	13 (22.4)		55 (12.2)	76 (16.9)	
Missing^d^	0 (0)	1 (1.7)		7 (1.6)	5 (1.1)	
**Alcohol intake median (range)**						
Grams/day	2.82 (0–17.9)	2.74 (0–19.2)	0.7	2.20 (0–30.6)	2.22 (0–27.9)	0.9
Missing^d^ n (%)	7 (12.07)	7 (12.07)		59 (13.11)	57 (12.67)	
**Physical activity n (%)**						
Inactive	8 (13.79)	9 (15.52)	0.3	83 18.44	75 (16.67)	0.9
Moderately inactive	19 (32.76)	11 (18.97)		135 (30.00)	144 (32.00)	
Moderately active	18 (31.04)	18 (31.03)		111 (24.67)	109 (24.22)	
Active	8 (13.79)	14 (24.14)		63 (14.00)	64 (14.22)	
Missing^d^	5 (8.62)	6 (10.34)		58 (12.89)	58 (12.89)	

^a^Includes complete case–control sets.

^b^Paired Wilcoxon signed rank test for continuous variables, Chi-Square tests for categorical variables.

^c^Includes 38 case control pairs with follow up samples.

^d^Missing category not included in the statistical comparisons.

### Tumor characteristics

Among the cases, there were some important differences between the discovery set and the validation set. First, in the discovery dataset almost half (46.6%) of the cases were rectal cancers compared to 34.7% in the validation set. In addition, a higher proportion of the discovery set cases were stage IV (22.4%) compared to 14.2% in the validation set (Supplementary Table S3).

### Biomarker identification and selection

**Figure 1:**
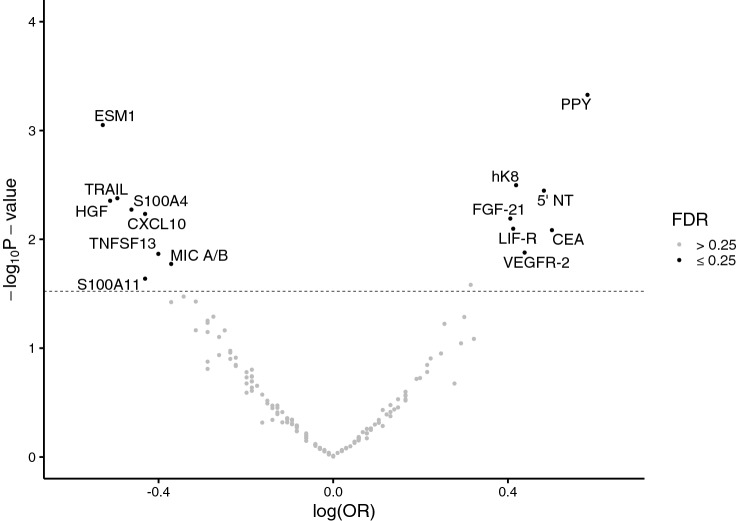
Volcano plot depicting results from the discovery phase of the study. Odds ratios were calculated using conditional logistic regression based on individuals’ baseline- and repeated values, separated in time. The model was adjusted for smoking status, BMI, and level of education. The dotted line represents FDR = 0.25.

After preprocessing and exclusions of proteins not passing quality control, 160 proteins remained. In our initial analyses, 15 proteins were associated with colorectal cancer at an FDR cutoff of 0.25 (Fig. 1, Table 2). Of these, we selected 13 and excluded two proteins (TNFSF13 and S100A4). TNFSF13 was excluded in favor of MIC A/B based on literature review, and S100A4 was excluded as it failed quality control in the designing of the custom multiplex panel. We also included eight additional proteins previously reported to be associated with colorectal cancer risk in the prediagnostic setting^10,22,23,25^.

**Table 2:**
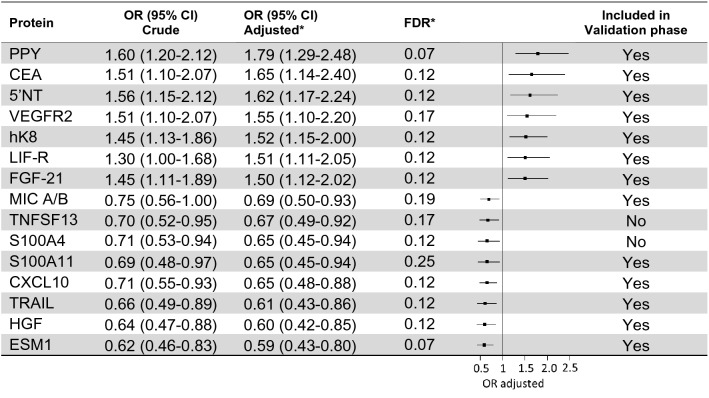
Top 15 proteins identified in the discovery phase.

*Adjusted for BMI, smoking and education and conditioned on matching criteria (age, sex and sampling date).

In the discovery analysis, PPY showed the strongest overall association with colorectal cancer with an OR of 1.79 (95% CI 1.29–2.48) per 1 SD. In total, seven proteins had higher levels in cases compared to controls and eight had lower levels (Fig. 1). Aside from PPY, top proteins included CEA (OR: 1.65, 95% CI 1.14–2.40) and 5’NT (OR: 1.62, 95% CI 1.17–2.24). ESM1 was the protein with the strongest inverse association with colorectal cancer (OR: 0.59 95% CI 0.43–0.80), closely followed by HGF (OR: 0.60 95% CI 0.42–0.85).

In analyses rerun after excluding two case–control pairs with identity mismatch 12 out of the 13 originally selected proteins remained among the top hits, and an additional six potentially significant proteins were identified (Supplementary Fig. S1, Supplementary Table S4). Although the stage of the validation analyses prevented their inclusion in the custom panel, we chose to present the results for potential future replication.

### Custom panel quality control

For the custom multiplex panel the average intra-assay CV was 7% and the average inter-assay CV was 12%. Three proteins had an intra-assay CV between 5–10%, no proteins had intra CV values above 10%. For the inter-assay CV, 17 proteins had an inter-assay CV value between 10 and 20% and one protein had inter-assay CV values between 20 and 30%. The singleplex CEA assay had an intra-assay CV of 11% and inter-assay CV of 20%.

The 21 proteins included in the custom panel (Supplementary Table S2) were analyzed in a validation set consisting of 1000 samples from 461 case–control pairs (with 39 pairs including repeated samples from both cases and controls). Of these, 12 samples failed quality control and were excluded, together with their matched cases or controls, from downstream analyses (Supplementary Fig. S2). The final validation set thus included 450 complete case–control pairs, of which 38 pairs had repeated measurements.

### Main analyses in the validation set

No proteins were associated with colorectal cancer risk at significance level *p* < 0.05. (Table 3). Out of the 13 proteins selected from the dicovery set, seven retained the same direction of association although with varying degrees of attenuation. The strongest positive association was for FGF-21 (OR: 1.14, 95% CI 0.99–1.30), followed by CEA (OR: 1.10, 95% CI 0.95–1.28) and PPY (OR: 1.08, 95% CI 0.93–1.26), all showing the same direction of association as previously identified. Only minimal differences in point estimates were observed when comparing models with imputed covariate data to models based only on collected data (data not shown).

**Table 3:**
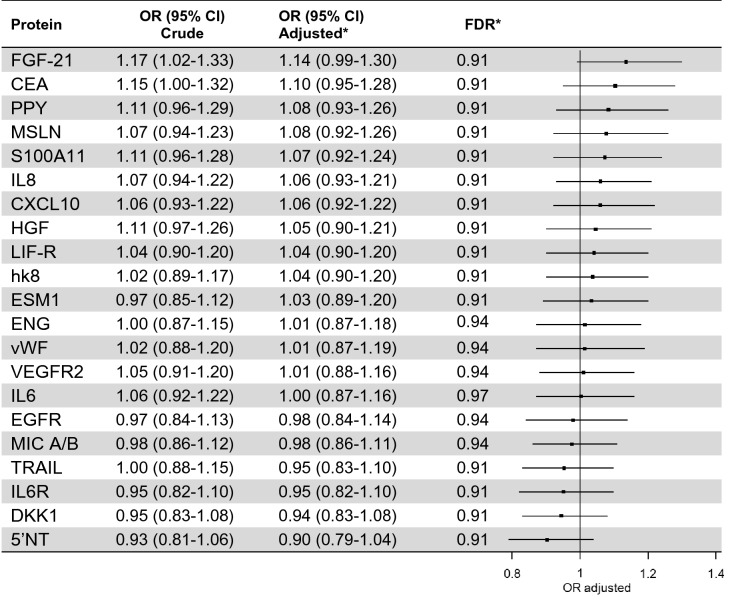
Proteins included in the validation phase main analysis.

*Adjusted for BMI, smoking and education and conditioned on matching criteria (age, sex and sampling date).

### Subgroup analyses in the validation set

**Figure 2:**
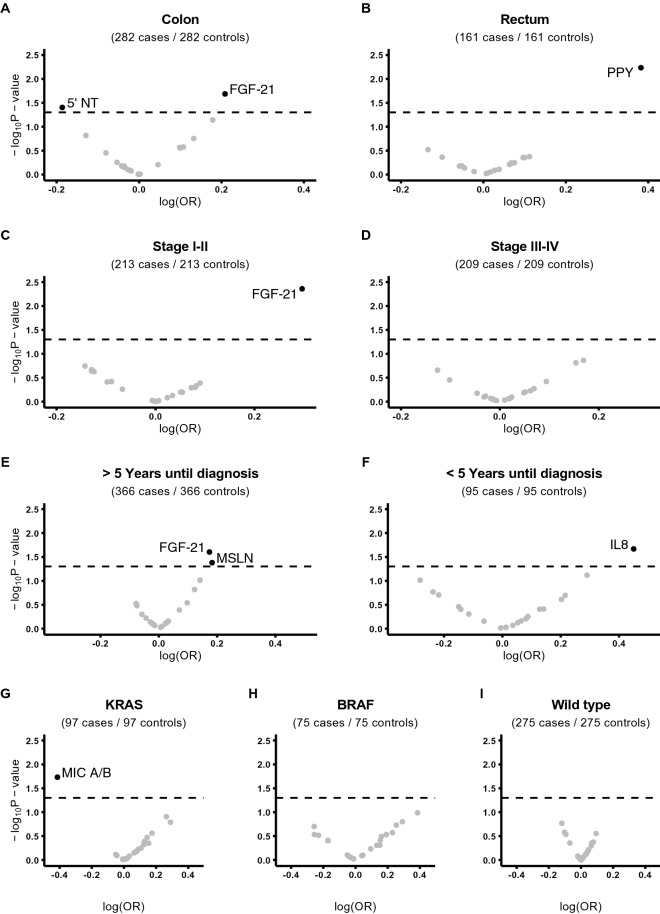
Volcano plots for stratified analyses comparing Colon and Rectum (**A**,**B**), Stage I–II and Stage III–IV (**C**,**D**), proximity to diagnosis (**E**,**F**) and molecular subtypes (**G**–**I**). All models were adjusted for smoking status, BMI, and level of education. The dotted line marks a *p* value cutoff of 0.05.

Stratified subgroup analyses were performed to take differences in tumor location, stage, time to diagnosis and molecular tumor subtypes into account. Results from these analyses are presented in Fig. 2 and Supplementary Table S5. In the subgroup analyses based on tumor site, FGF-21 (OR: 1.23, 95% CI 1.03–1.47) and 5’NT (OR: 0.86, 95% CI 0.79–0.99), were associated with colon but not rectal cancer, whereas PPY (OR: 1.47, 95% CI 1.12–1.92) was associated with rectal but not colon cancer. FGF-21 also retained statistical significance in stage I–II colorectal cancer and in cases with samples collected more than 5 years before diagnosis. No proteins showed statistical significance in the analyses including only stage III–IV colorectal cancer. In analyses stratified for molecular subtypes, MIC A/B was associated with a lower risk of *KRAS*-mutated colorectal cancer (OR: 0.66, 95% CI 0.47–0.93), no proteins were associated with the risk of *BRAF*-mutated or *KRAS-BRAF*-wild type colorectal cancer.

### Lasso regression in the validation set

**Figure 3:**
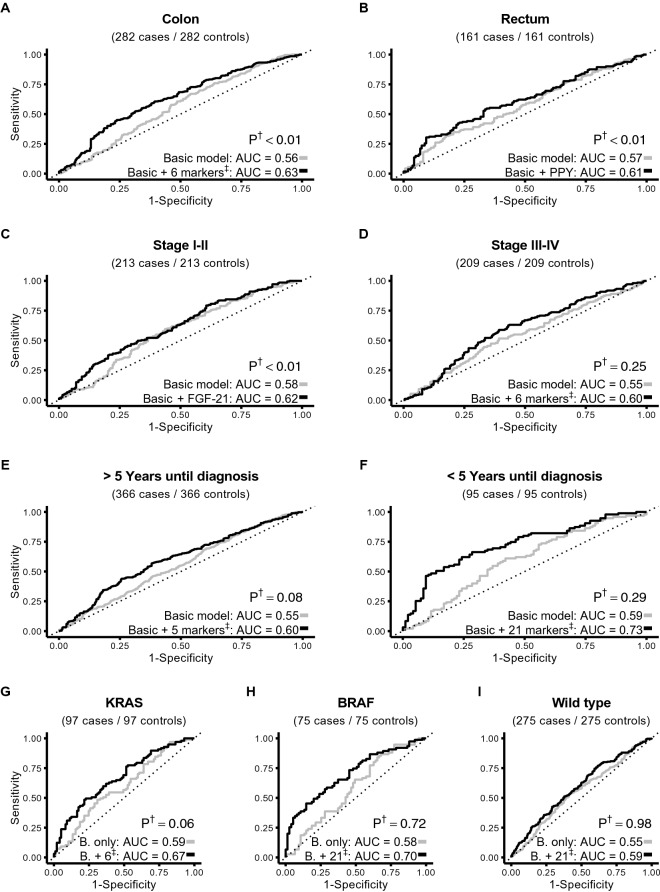
ROC curves for stratified analyses comparing Colon and Rectum (**A**,**B**), Stage I–II and Stage III–IV (**C**,**D**), years until diagnosis (**E**,**F**) and molecular subtypes (**G**–**I**). All models were adjusted for smoking status, BMI, level of education, age and sex. For complete protein panels see Supplementary Table S5. ^†^Based on the loglikelihood ratio test between the two models. ^‡^Selected proteins are listed in Supplementary Table S6.

In order to determine if any specific combination of proteins could predict colorectal cancer risk better than individual proteins, we used a Lasso logistic regression model. Models containing only the risk factors included as covariates in the conditional logistic regression analyses (age, sex, BMI, smoking status and level of education) were compared to our Lasso-generated protein models using ROC curves, both for the main analysis (Supplementary Fig. S3) and for the subgroup analyses (Fig. 3). In the main model, FGF-21 was the only protein with sufficient predictive ability to be included, increasing the AUC slightly from 0.55 (95% CI 0.51–0.59) in the risk-factor-only model to 0.57 (95% CI 0.50–0.53). The likelihood ratio test indicated that the increase was borderline significant (*p* = 0.046). In the subgroup analyses (Fig. 3), the highest discriminative ability was seen for colon cancer, for which the AUC increased from 0.56 (95% CI 0.52–0.61) in the risk-factor-only model to 0.63 (95% CI 0.59–0.68), *p* = 0.003 and for individuals with stage I–II colorectal cancer, for which the AUC increased from 0.58 (95% CI 0.53–0.64) to 0.62 (95% CI 0.57–0.67), *p* = 0.002.

### Linear mixed models

No statistically significant differences were found for either FGF-21 or PPY on the 38 case sets with repeated samples (Supplementary Fig. S4).

## Discussion

Utilizing a two-tiered approach with prospectively collected samples, we aimed to identify protein biomarkers related to colorectal cancer susceptibility or early diagnosis. In the discovery phase, we identified 15 proteins with significantly altered levels in colorectal cancer cases compared to controls, of which 13 were then selected for further analysis. None of the selected proteins retained statistical significance in the main validation analysis, but two proteins of particular interest, FGF-21 and PPY, were identified when stratifying analyses by tumor location, stage and time to diagnosis.

Multiple studies have aimed to identify protein biomarkers that could be utilized for precision screening for early detection of colorectal cancer^5,12,23^. However, the majority of these have used samples from either a clinical or a screening setting. These study designs, although successful in identifying biomarkers or biomarker combinations with relatively good predictive ability (AUC > 0.7), may not be useful for identifying prospective patients years, or even months, before diagnosis. Clearly, identifying markers that are measurable before diagnosis, to be used for refined risk stratification or even early diagnosis has proven difficult. Very few studies have utilized prospectively collected samples, and results have proven hard to replicate^12^. Because most previous studies did not include prospective samples, it is difficult to compare the results from our study to those of others. The importance of conducting additional studies in a prediagnostic, asymptomatic setting, and not only in cancer patients or screening participants therefore needs to be emphasized.

In our study, which focused mainly on inflammatory targets, FGF-21 was the protein that performed best throughout all stages. However, it did not reach significance in the full dataset combining both colon and rectal cancer cases. Interestingly, when stratifying by anatomical tumor sub-site, it became evident that the association between FGF-21 and colon cancer was driving this association. FGF-21 remained associated with colon cancer after adjusting for BMI, education and smoking. Notably, FGF-21 was associated with early, but not late, stages of colorectal cancer. We previously identified FGF-21 as associated with colorectal cancer risk in a study investigating potential protein biomarkers of metabolic syndrome^13^, where we found it to be strongly associated with BMI and therefore of potential interest as a colorectal cancer screening biomarker. FGF-21 has since been shown to associate with colorectal cancer in at least one other study^26^, where it was reported to be positively associated with both early and late stage colorectal cancers.

In the rectal cancer group, pancreatic prohormone (PPY), also known as pancreatic polypeptide^27^, was the most prominent finding. This protein is mainly produced in the pancreas and secreted postprandially where it slows down the digestive process^28^. PPY is a marker of some pancreatic tumors including pancreatic polypeptide-secreting tumor of the distal pancreas (PPoma) and Multiple endocrine neoplasia type 1 (MEN1) both of which are characterized by high serum levels of PPY^29^. Few studies have specifically examined levels of PPY in colorectal cancer patients compared to healthy controls. One small-scale study from Poland, including 60 colorectal cancer patients and 30 healthy controls, found elevated levels of PPY in colon cancer patients compared to rectal cancer patients and cancer free controls^30^.

Despite being potentially predictive and possibly related to colorectal cancer etiology, neither FGF-21 nor PPY would be useful as standalone biomarkers of colon or rectal cancer. We therefore explored the possibility of combining different protein markers in order to identify a panel with better discriminative capabilities, an approach that has been proven successful in similar study designs^10,11,31,32^. However, the addition of the top markers selected by Lasso regression to conventional risk factors increased the predictive ability only modestly from an AUC of 0.55–0.57, still far from clinically useful. Subgroup analyses resulted in larger improvements in discriminative ability, although all AUCs remained below 0.7. It should be noted that the predictive performance of our basic model was quite low compared to previous studies^10,11,23,32^. Possible explanations may include lack of family history data in our study, and the low ages at sampling and at colorectal cancer diagnosis (mean of < 70 years in both data sets) due to the recruitment protocol with ongoing sampling at defined ages (40, 50 and 60 years).

Aside from the markers identified in our discovery phase the custom panel also included eight proteins selected from previous promising findings and available for Olink multiplexing, namely Cohen et al.^22^ and Rho et al.^23^. However, none were significantly associated with colorectal cancer in our study population. In Cohen et al. the authors developed a general blood based test for cancer detection, which was tested in a set of cancer patients and controls. Although the test does not target colorectal cancer specifically, potential colorectal cancer biomarkers were evaluated. Of the additional five markers from Cohen et al. selected for our study, none were included in the final CancerSEEK panel. The only colorectal cancer marker in the CancerSEEK panel was CEA, which also reached statistical significance in our discovery dataset and was thus already selected for our custom array. CEA is used in clinical practice to follow colorectal cancer patients^33^.

In contrast to Cohen et al., and Rho et al.^23^ conducted a study using prediagnostic blood samples and four biomarkers of particular interest for early detection of colorectal cancer (BAG4, IL6R/ST, VWF and EGFR). One of our initial aims was to try to replicate all four of these markers, but we were limited by the proteins available on the Olink panels and therefore could not include BAG4. The lack of association with colorectal cancer for the other three markers in our study may be due to the longer time between sample donation and cancer diagnosis in our population compared to Rho et al. in which it was less than 3 years. Another reason may be that our study lacked power to detect small effect sizes, even if there were differences between prospective cases and controls more than 3 years before case diagnosis.

Our study has several strengths including the prospective approach, the two independent sample sets and the large size of the validation set. However, several limitations need to be addressed. First, the composition of the cases in the two datasets differed with respect to clinical characteristics. Rectal cancer and stage IV cancer were more common in the discovery set compared to the validation set. These discrepancies primarily reflect the small samples size in the discovery set, which was a conscious trade-off to allow the stringent selection of cases and controls with time-matched, repeated prediagnostic samples. The discovery data set is, therefore, not entirely representative of the site and stage proportions in the general population in Västerbotten, which is captured in the larger validation dataset. Since the result for FGF-21 was retained for colon cancer and stage I-II colorectal cancer in the validation dataset, the clinical differences between the two datasets do not seem to have affected the main findings. The comparatively low proportion of stage III-IV cancers in the validation set could probably explain, at least partly, why CEA did not reach significance despite being one of few commonly used biomarkers for colorectal cancer monitoring^34^. In addition, we lacked information on family history, which is known to be one of the best predictors of future colorectal cancer risk^7^ and likely would have improved clinical risk prediction models. Furthermore, we chose to rely mainly on statistical cut-offs for marker selection when proceeding from the discovery to the validation stage on the study. However, the small size of our discovery dataset may have hindered the identification of true colorectal cancer biomarkers. In hindsight an approach combining statistical cut-offs with biological relevance might have resulted in more markers being validated. Finally, for sample size reasons, Lasso regression to select protein markers was performed without dividing our dataset into a training and testing set and results are therefore in need of further validation.

Our findings highlight the challenge of identifying cancer biomarkers that can be used in the pre-diagnostic window of opportunity for early detection. For risk stratification, with the vision of achieving effective precision screening, single biomarkers or small marker panels may not be sufficient. Instead, we would advocate also attempting to identify patterns, based on panels of biomarkers, which might achieve more precise risk stratification. For translation to commercially viable blood tests, the composition and size of biomarker panels will require consideration of cost effectiveness, such as numbers needed to prevent one colorectal cancer case or colorectal cancer death.

In conclusion, we identified two markers (FGF-21 and PPY) that were associated with colon and rectal cancer respectively, suggesting a potential of biomarkers to discriminate between different subtypes of colorectal cancer. Approaches for future studies of colorectal cancer risk prediction and early detection biomarkers should probably focus on large collections of prospectively collected samples and deeply phenotyped colorectal cancer cases, and perhaps use machine learning on high-dimensional biomarker platforms to identify biomarker risk patterns.

## Supplementary Information

Supplementary Information

## Data Availability

The datasets generated and/or analyzed during the current study are considered personal data, which prohibits us from storing them in a public depository. However, all data are archived at the Biobank Research Unit at Umeå University, and access for secondary use can be granted conditional upon meeting Swedish requirements for human research.
