# Supplementary material for: A two-tiered targeted proteomics approach to identify pre-diagnostic biomarkers of colorectal cancer risk
Source: Sci Rep. 2021 Mar 4;11:5151. doi: 10.1038/s41598-021-83968-6 (PMC7933352; doi:10.1038/s41598-021-83968-6)
Supplement: Supplementary file 1 — Supplementary Information [file 41598_2021_83968_MOESM1_ESM.pdf]

Supplemental Material

A two-tiered targeted proteomics approach to identify pre-diagnostic biomarkers of colorectal cancer risk

Sophia Harlid*, Justin Harbs, Robin Myte, Carl Brunius, Marc J. Gunter, Richard Palmqvist Xijia Liu and Bethany Van Guelpen

***Correspondence to**: Sophia Harlid

Table of Contents: Page

Table of Contents:	Page
Figure S1 (Discovery phase 2, comparison analysis)	2
Figure S2 (Flowchart)	3
Figure S3 (Lasso regression, main model)	4
Figure S4 (Mixed models)	5
Table S1 (Proteins included in Proseek Multiplex® immunoassay panels)	6
Table S2 (Custom panel)	11
Table S3 (Tumor characteristics)	12
Table S4 (Discovery phase 2, comparison analysis)	13
Table S5 (Subgroup analyses)	14
Table S6 (Proteins selected by Lasso regression models)	19
References	20

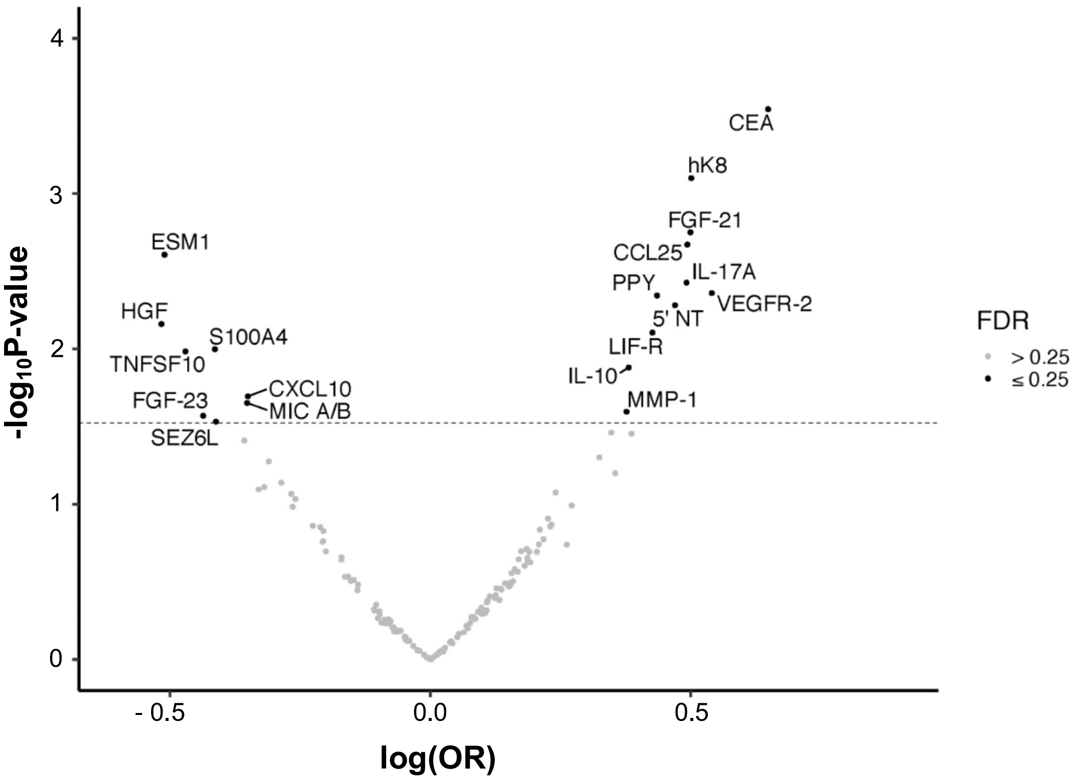

Figure S1. Volcano plot depicting re-analysis of the 160 protein markers *after exclusions of samples with identity mismatch* [1]. The dashed line indicates the FDR cut off value of 0.25.

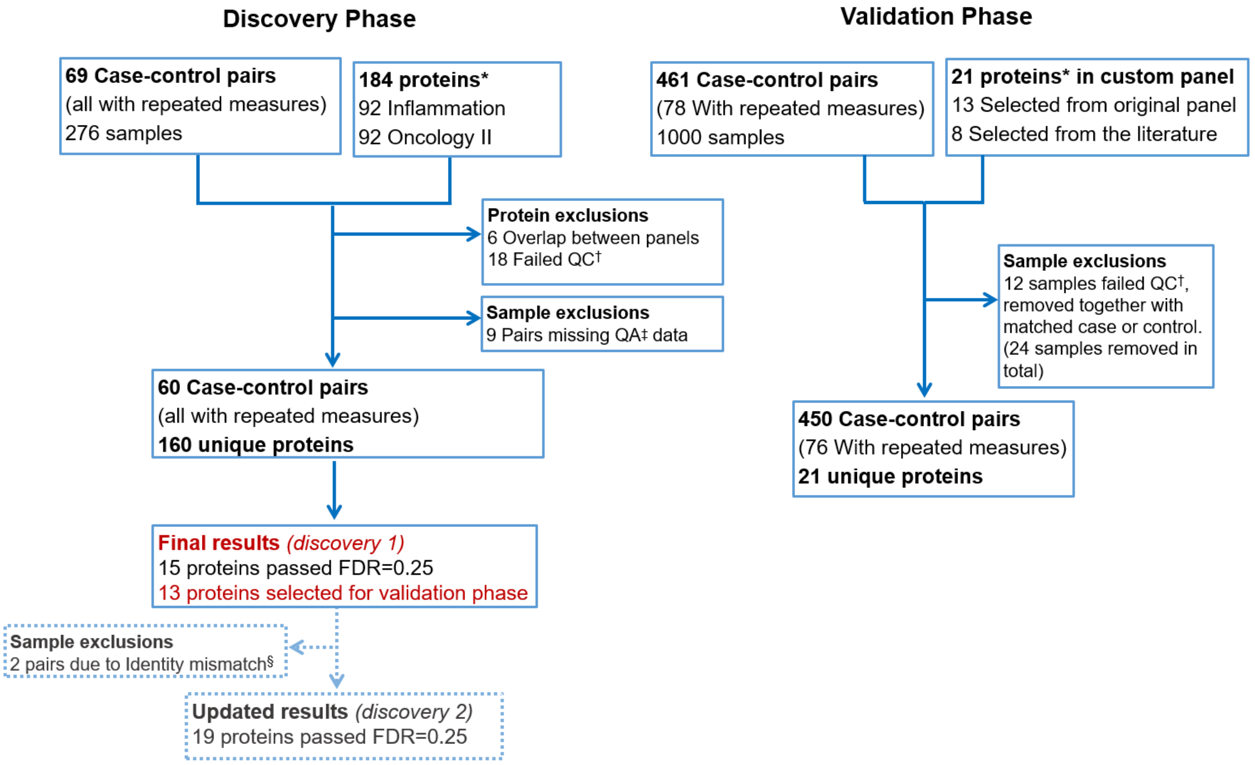

*Proteins analyzed by Olink proteomics (Inflammation and Oncology II panels) using Proximity Extension Assay (PEA) technology
†QC: Quality Control
‡QA: Questionnaire
§Repeated samples had been wrongly attributed to the same individual

Figure S2. Flowchart showing sample and protein selection in the discovery and validation phases of the project.

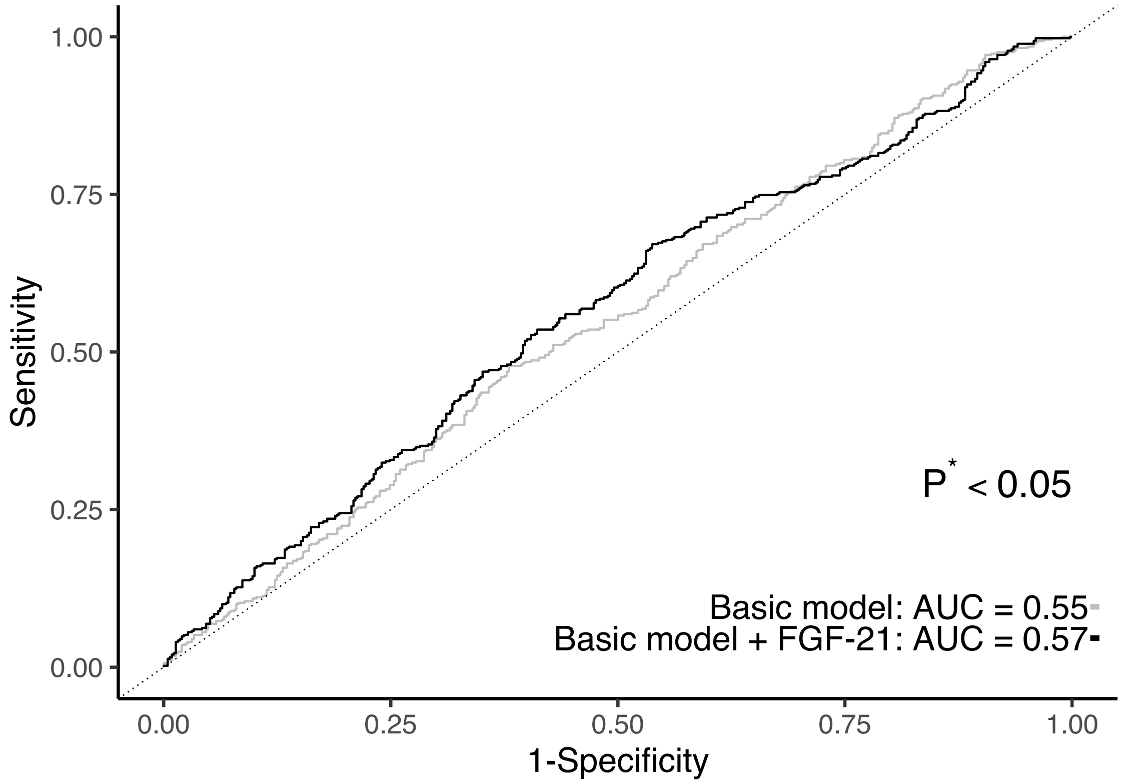

* Based on the loglikelihood ratio test between the two models.

Figure S3. Receiver operator curves (ROC) showing two different models. Model 1 (grey line) includes only the covariates included in the logistic regression analyses (age, sex, BMI, smoking status, level of education, age and sex) and Model 2 (black line) includes the addition of FGF-21, which was the only protein selected by the Least absolute shrinkage and selection operator, Lasso, combined with logistic regression.

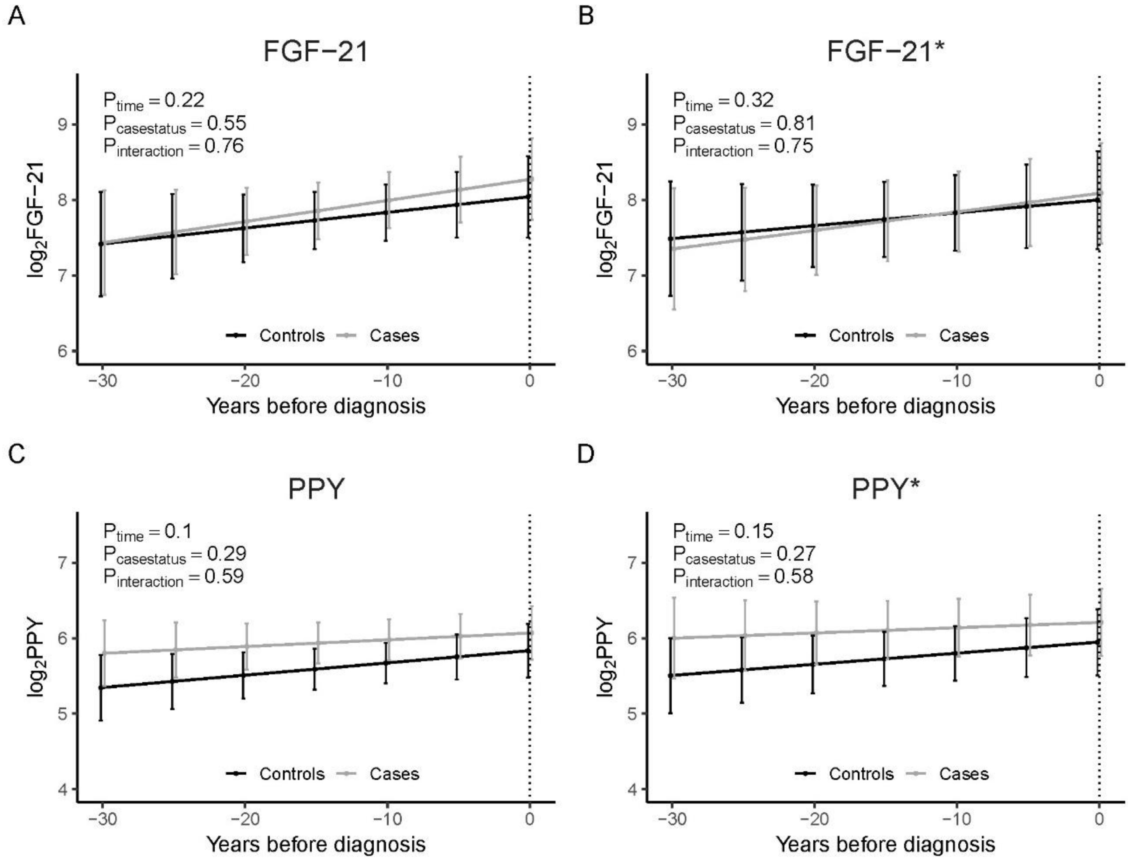

Figure S4. Changes in FGF-21 and PPY concentrations over time in 38 CRC cases and 38 matched controls from the discovery set with repeated measurements before case diagnosis. Linear mixed models were used to estimate marginal effects and 95% CIs of time. All models included case-control pair as random factors. Models (A) and (C) included time until case diagnosis, case–control status as fixed factors. Models (B) and (D) additionally smoking status, educational level, and BMI as fixed factors.

Table S1. Proteins included in Proseek Multiplex® immunoassay panels

Protein	UniProt No	Immunoassay panel	% samples < LOD
Hepatocyte growth factor (HGF)	P14210	Both	0
Interleukin-6 (IL-6)	P05231	Both	0
Stem cell factor (SCF)	P21583	Both	0
TNF-related apoptosis-inducing ligand (TRAIL)	P50591	Both	0
Transforming growth factor alpha (TGF-alpha)	P01135	Both	0
Vascular endothelial growth factor A (VEGF-A)	P15692	Both	0
Adenosine Deaminase (ADA)	P00813	Inflammation	0
Artemin (ARTN)	Q5T4W7	Inflammation	91
Axin-1 (AXIN1)	O15169	Inflammation	0
Beta-nerve growth factor (Beta-NGF) P01138	P01138	Inflammation	0
Brain-derived neurotrophic factor (BDNF)	P23560	Inflammation	19
Caspase 8 (CASP-8)	Q14790	Inflammation	0
C-C motif chemokine 19 (CCL19)	Q99731	Inflammation	0
C-C motif chemokine 20 (CCL20)	P78556	Inflammation	0
C-C motif chemokine 23 (CCL23)	P55773	Inflammation	0
C-C motif chemokine 25 (CCL25)	O15444	Inflammation	0
C-C motif chemokine 28 (CCL28)	Q9NRJ3	Inflammation	0
C-C motif chemokine 4 (CCL4)	P13236	Inflammation	0
CD40L receptor (CD40)	P25942	Inflammation	0
CUB domain-containing protein 1 (CDCP1)	Q9H5V8	Inflammation	0
C-X-C motif chemokine 1 (CXCL1)	P09341	Inflammation	0
C-X-C motif chemokine 10 (CXCL10)	P02778	Inflammation	0
C-X-C motif chemokine 11 (CXCL11)	O14625	Inflammation	0
C-X-C motif chemokine 5 (CXCL5)	P42830	Inflammation	0
C-X-C motif chemokine 6 (CXCL6)	P80162	Inflammation	0
C-X-C motif chemokine 9 (CXCL9)	Q07325	Inflammation	0
Cystatin D (CST5)	P28325	Inflammation	0
Delta and Notch-like epidermal growth factor-related recep (DNER)	Q8NFT8	Inflammation	0
Eotaxin-1 (CCL11)	P51671	Inflammation	0
Eukaryotic translation initiation factor 4E-binding protein 1 (4E-BP1)	Q13541	Inflammation	0
Fibroblast growth factor 19 (FGF-19)	O95750	Inflammation	0
Fibroblast growth factor 21 (FGF-21)	Q9NSA1	Inflammation	0
Fibroblast growth factor 23 (FGF-23)	Q9GZV9	Inflammation	0
Fibroblast growth factor 5 (FGF-5)	Q8NF90	Inflammation	1
Fms-related tyrosine kinase 3 ligand (Flt3L)	P49771	Inflammation	0
Fractalkine (CX3CL1)	P78423	Inflammation	0
Glial cell line-derived neurotrophic factor (hGDNF)	P39905	Inflammation	0

Table continues on next page

Table S1. Cont.

**Protein**	**UniProt No**	**Immunoassay panel**	**% samples < LOD**
Interferon gamma (IFN-gamma)	P01579	Inflammation	95
Interleukin-1 alpha (IL-1 alpha)	P01583	Inflammation	94
Interleukin-10 (IL-10)	P22301	Inflammation	0
Interleukin-10 receptor subunit alpha (IL-10RA)	Q13651	Inflammation	55
Interleukin-10 receptor subunit beta (IL-10RB)	Q08334	Inflammation	0
Interleukin-12 subunit beta (IL-12B)	P29460	Inflammation	0
Interleukin-13 (IL-13)	P35225	Inflammation	81
Interleukin-15 receptor subunit alpha (IL-15RA)	Q13261	Inflammation	0
Interleukin-17A (IL-17A)	Q16552	Inflammation	32
Interleukin-17C (IL-17C)	Q9P0M4	Inflammation	23
Interleukin-18 (IL-18)	Q14116	Inflammation	0
Interleukin-18 receptor 1 (IL-18R1)	Q13478	Inflammation	0
Interleukin-2 (IL-2)	P60568	Inflammation	99
Interleukin-2 receptor subunit beta (IL-2RB)	P14784	Inflammation	86
Interleukin-20 (IL-20)	Q9NYY1	Inflammation	61
Interleukin-20 receptor subunit alpha (IL-20RA)	Q9UHF4	Inflammation	85
Interleukin-22 receptor subunit alpha-1 (IL-22 RA1)	Q8N6P7	Inflammation	99
Interleukin-24 (IL-24)	Q13007	Inflammation	90
Interleukin-33 (IL-33)	O95760	Inflammation	99
Interleukin-4 (IL-4)	P05112	Inflammation	70
Interleukin-5 (IL-5)	P05113	Inflammation	56
Interleukin-7 (IL-7)	P13232	Inflammation	0
Interleukin-8 (IL-8)	P10145	Inflammation	0
Latency-associated peptide transforming growth factor beta 1 (LAP TGF-beta-1)	P01137	Inflammation	0
Leukemia inhibitory factor (LIF)	P15018	Inflammation	86
Leukemia inhibitory factor receptor (LIF-R)	P42702	Inflammation	0
Macrophage colony-stimulating factor 1 (CSF-1)	P09603	Inflammation	0
Macrophage inflammatory protein 1-alpha (MIP-1 alpha, CCL3)	P10147	Inflammation	0
Matrix metalloproteinase-1 (MMP-1)	P03956	Inflammation	0
Matrix metalloproteinase-10 (MMP-10)	P09238	Inflammation	0
Monocyte chemotactic protein 1 (MCP-1)	P13500	Inflammation	0
Monocyte chemotactic protein 2 (MCP-2, 8CCL89)	P80075	Inflammation	0
Monocyte chemotactic protein 3 (MCP-3)	P80098	Inflammation	17
Monocyte chemotactic protein 4 (MCP-4)	Q99616	Inflammation	0
Natural killer cell receptor 2B4 (BDNF4)	Q9BZW8	Inflammation	0
Neurotrophin-3 (NT-3)	P20783	Inflammation	0
Neurturin (NRTN)	Q99748	Inflammation	94

Table continues on next page

Table S1. Cont.

Protein	UniProt No	Immunoassay panel	% samples < LOD
Oncostatin-M (OSM)	P13725	Inflammation	0
Osteoprotegerin (OPG)	O00300	Inflammation	0
Programmed cell death 1 ligand 1 (PD-L1)	Q9NZQ7	Inflammation	0
Protein S100-A12 (EN-RAGE)	P80511	Inflammation	0
Signaling lymphocytic activation molecule (SLAMF1)	Q13291	Inflammation	0
SIR2-like protein 2 (SIRT2)	Q8IXJ6	Inflammation	0
STAM-binding protein (STAMPB)	O95630	Inflammation	0
Sulfotransferase 1A1 (ST1A1)	P50225	Inflammation	1
T cell surface glycoprotein CD6 isoform (CD6)	Q8WWJ7	Inflammation	0
T-cell surface glycoprotein CD5 (CD5)	P06127	Inflammation	0
Thymic stromal lymphopoietin (TSLP)	Q969D9	Inflammation	99
TNF-beta (TNFB)	P01374	Inflammation	0
TNF-related activation-induced cytokine (TRANCE)	O14788	Inflammation	0
Tumor necrosis factor (Ligand) superfamily, member 12 (TWEAK)	Q4ACW9	Inflammation	0
Tumor necrosis factor (TNF)	P01375	Inflammation	87
Tumor necrosis factor ligand superfamily member 14 (TNFSF14)	O43557	Inflammation	0
Tumor necrosis factor receptor superfamily member 9 (TNFRSF9)	Q07011	Inflammation	0
Urokinase-type plasminogen activator (uPA)	P00749	Inflammation	0
5'-nucleotidase (5'-NT)	P21589	Oncology II	0
A disintegrin and metalloproteinase with thrombospondin motifs 15 (ADAM-TS 15)	Q8TE58	Oncology II	0
A/B (MIC-A/B)	Q29980 Q29983	Oncology II	2
AlPha-taxilin (TXLNA)	P40222	Oncology II	0
AmPhiregulin (AR)	P15514	Oncology II	0
Annexin A1 (ANXA1)	P04083	Oncology II	45
Carbonic anhydrase 9 (CA9)	Q16790	Oncology II	0
Carboxypeptidase E (CPE)	P16870	Oncology II	0
Carcinoembryonic antigen-related celladhesion molecule 1 (CEACAM1)	P13688	Oncology II	0
Carcinoembryonic antigen-related celladhesion molecule 5 (CEACAM5)	P06731	Oncology II	9
Cathepsin L2 (CTSV)	O60911	Oncology II	0
CD160 antigen (CD160)	O95971	Oncology II	0
CD27 antigen (CD27)	P26842	Oncology II	0
CD48 antigen (CD48)	P09326	Oncology II	0
CD70 antigen (CD70)	P32970	Oncology II	0
Cornulin (CRNN)	Q9UBG3	Oncology II	24
C-type lectin domain family 4 member K(CD207)	Q9UJ71	Oncology II	0
C-X-C motif chemokine 13 (CXCL13)	O43927	Oncology II	0
Cyclin-dependent kinase inhibitor 1 (CDKN1A)	P38936	Oncology II	0

Table continues on next page

Table S1. Cont.

Protein	UniProt No	Immunoassay panel	% samples < LOD
Delta-like protein 1 (DLL1)	O00548	Oncology II	0
Disintegrin and metalloproteinase domain-containing protein (ADAM8)	P78325	Oncology II	0
Endothelial cell-specific molecule 1 (ESM-1)	Q9NQ30	Oncology II	0
EPhrin type-A receptor 2 (EPHA2)	P29317	Oncology II	0
FAS-associated death domain protein (FADD)	Q13158	Oncology II	0
Fc receptor-like B (FCRLB)	Q6BAA4	Oncology II	0
Fibroblast growth factor-binding Protein 1(FGF-BP1)	Q14512	Oncology II	0
Folate receptor alpha (FR-alpha)	P15328	Oncology II	0
Folate receptor gamma (FR-gamma)	P41439	Oncology II	0
Furin (FURIN)	P09958	Oncology II	0
Galectin-1 (Gal-1)	P09382	Oncology II	0
Glypican-1 (GPC1)	P35052	Oncology II	0
Granzyme B (GZMB)	P10144	Oncology II	0
Granzyme H (GZMH)	P20718	Oncology II	0
ICOS ligand (ICOSLG)	O75144	Oncology II	0
Insulin-like growth factor 1 receptor (IGF1R)	P08069	Oncology II	0
Integrin alpha-V (ITGAV)	P06756	Oncology II	0
Integrin beta-5 (ITGB5)	P18084	Oncology II	0
Interferon gamma receptor 1 (IFN-gamma-R1)	P15260	Oncology II	0
Kallikrein-11 (hK11)	Q9UBX7	Oncology II	0
Kallikrein-13 (KLK13)	Q9UKR3	Oncology II	0
Kallikrein-14 (hK14)	Q9P0G3	Oncology II	0
Kallikrein-8 (hK8)	O60259	Oncology II	0
Ly6/PLAUR domain-containing Protein 3 (LYPD3)	O95274	Oncology II	0
Melanoma-derived growth regulatory Protein (MIA)	Q16674	Oncology II	0
Mesothelin (MSLN)	Q13421	Oncology II	0
Methionine aminoPeptidase 2 (MetAp2)	P50579	Oncology II	0
Midkine (Mk)	P21741	Oncology II	0
Mothers against decapentaplegic homolog 5 (MAD homolog 5)	Q99717	Oncology II	0
Mucin-16 (MUC-16)	Q8WXI7	Oncology II	0
Nectin-4 (PVRL4)	Q96NY8	Oncology II	0
Pancreatic prohormone (PPY)	P01298	Oncology II	0
Podocalyxin (PODXL)	O00592	Oncology II	0
Pro-epidermal growth factor (EGF)	P01133	Oncology II	0
Protein CYR61 (CYR61)	O00622	Oncology II	0
Protein S100-A11 (S100A11)	P31949	Oncology II	0
Protein S100-A4 (S100A4)	P26447	Oncology II	0

Table continues on next page

Table S1. Cont.

Protein	UniProt No	Immunoassay panel	% samples < LOD
Proto-oncogene tyrosine-protein kinasereceptor Ret (RET)	P07949	Oncology II	0
Receptor tyrosineprotein kinase erbB-2(ERBB2)	P04626	Oncology II	0
Receptor tyrosineprotein kinase erbB-3 (ERBB3)	P21860	Oncology II	0
Receptor tyrosineprotein kinase erbB-4 (ERBB4)	Q15303	Oncology II	0
R-spondin-3 (RSPO3)	Q9BXY4	Oncology II	0
Secretory carrier-associated membraneprotein 3 (SCAMP3)	O14828	Oncology II	0
Seizure 6-like protein (SEZ6L)	Q9BYH1	Oncology II	0
SPARC (SPARC)	P09486	Oncology II	0
Syndecan-1 (SYND1)	P18827	Oncology II	0
T-cell leukemia / lymphoma Protein 1A (TCL1A)	P56279	Oncology II	0
TGF-beta receptor type-2 (TGFR-2)	P37173	Oncology II	0
Tissue factor pathway inhibitor 2 (TFP1-2)	P48307	Oncology II	0
T-lymphocyte surface antigen Ly-9 (LY9)	Q9HBG7	Oncology II	0
Toll-like receptor 3 (TLR3)	O15455	Oncology II	0
Transmembrane glycoprotein NMB (GPNMB)	Q14956	Oncology II	0
Tumor necrosis factor ligand superfamilymember 13 (TNFSF13, APRIL)	O75888	Oncology II	0
Tumor necrosis factor ligand superfamilymember 6 (FASLG)	P48023	Oncology II	0
Tumor necrosis factor receptor superfamilymember 19 (TNFRSF19)	Q9NS68	Oncology II	0
Tumor necrosis factor receptor superfamilymember 4 (TNFRSF4)	P43489	Oncology II	0
Tumor necrosis factor receptor superfamilymember 6B (TNFRSF6B)	O95407	Oncology II	0
Tyrosineprotein kinase ABL1 (ABL1)	P00519	Oncology II	0
Tyrosineprotein kinase Lyn (LYN)	P07948	Oncology II	0
Vascular endothelial growth factor receptor 2 (VEGFR-2)	P35968	Oncology II	0
Vascular endothelial growth factor receptor 3 (VEGFR-3)	P35916	Oncology II	0
VEGF-co regulated chemokine 1 (CXCL17)	Q6UXB2	Oncology II	0
Vimentin (VIM)	P08670	Oncology II	0
WAP four-disulfide core domain protein 2 (WFDC2, HE4)	Q14508	Oncology II	0
Wnt inhibitory factor 1 (WIF-1)	Q9Y5W5	Oncology II	0
WNT1-inducible-signaling pathway protein 1 (WISP-1)	O95388	Oncology II	0
Xaa-pro aminopeptidase 2 (XPNPEP2)	O43895	Oncology II	0

Table S2. Proteins selected for inclusion in the custom panel

Protein	Origin	Full name	% samples < LOD
CEA*	Discovery phase	Carcinoembryonic antigen	38
hK8	Discovery phase	Kallikrein-8	0
FGF-21	Discovery phase	Fibroblast growth factor 21	0
ESM-1	Discovery phase	Endothelial cell-specific molecule 1	0
VEGFR2	Discovery phase	Vascular endothelial growth factor receptor 2	0
PPY	Discovery phase	Pancreatic polypeptide	0
5'NT	Discovery phase	5'-nucleotidase	0
HGF	Discovery phase	Hepatocyte growth factor	0
LIF-R	Discovery phase	Leukemia inhibitory factor receptor	0
TNFSF10/TRAIL	Discovery phase	TNF-related apoptosis-inducing ligand	0
CXCL10	Discovery phase	C-X-C motif chemokine 10	0
MIC A/B	Discovery phase	MHC class I polypeptide-related sequence A/B	2
S100A11	Replaced S100A4†	Protein S100-A11	0
IL-6RA	From literature [2]	Interleukin-6 receptor subunit alpha	0
vWF	From literature [2]	von Willebrand factor	0
EGFR	From literature [2]	Epidermal growth factor receptor	0
IL6	From literature [3]	Interleukin-6	0
IL8	From literature [3]	Interleukin-8	0
Dkk-1	From literature [3,4]	Dickkopf-related protein 1	0
MSLN	From literature [3,5]	Mesothelin	0
ENG	From literature [3]	Endoglin	0

*Run as uniplex due to problems in the custom panel design

† Replaced by S100A11 due to problems in the custom panel design.

Table S3. Clinical and molecular tumor characteristics of colorectal cancer patients included in the discovery and validation phases

Tumor characteristic		Discovery set (n=232) Cases (n=58)*	Validation set (n=976) Cases (n=450)
**Stage,** n (%)	I-II	23 (39.7)	213 (47.3)
	III-IV	29 (50.0)	209 (46.4)
	Missing	6 (10.3)	28 (6.2)
**Location,** n (%)	Proximal colon	11 (19.0)	140 (31.1)
	Distal colon	16 (27.6)	142 (31.6)
	Rectum	27 (46.6)	161 (35.8)
	Missing	4 (6.9)	7 (1.6)
**Molecular subtype,** n (%)	*KRAS* mut	-	97 (21.6)
	BRAF mut	-	75 (16.7)
	wild type	-	275 (61.1)
	Missing	-	3 (0.7)
**Follow-up**, n (%)			
Baseline sample	*>5 years*	58 (100.0)	366 (81.3)
	≤5 years	0 (0.0)	84 (18.7)
Repeat sample	*>5 years*	0 (0.0)	27 (6.0)
	≤5 years	58 (100.0)	11 (2.4)

*Individuals with identify mismatch are excluded from this table.

Table S4. Top 19 proteins identified in the discovery phase *2 (after individuals with identify mismatch were removed)*

Protein	OR (95% CI) Crude	OR (95% CI) Adjusted*	FDR*
CEA	1.74 (1.26-2.40)	1.91 (1.35-2.72)	0.05
VEGFR2	1.64 (1.15-2.33)	1.72 (1.18-2.49)	0.09
hk8	1.59 (1.21-2.08)	1.65 (1.23-2.21)	0.06
FGF-21	1.53 (1.15-2.05)	1.65 (1.20-2.25)	0.08
CCL25	1.57 (1.16-2.11)	1.64 (1.20-2.24)	0.08
IL17A	1.60 (1.16-2.22)	1.64 (1.17-2.28)	0.09
5'NT	1.60 (1.15-2.22)	1.60 (1.15-2.22)	0.09
PPY	1.53 (1.15-2.04)	1.55 (1.14-2.09)	0.09
LIF-R	1.44 (1.08-1.92)	1.53 (1.12-2.10)	0.11
IL10	1.38 (1.03-1.84)	1.46 (1.08-1.98)	0.15
MMP1	1.35 (1.00-1.83)	1.46 (1.05-2.03)	0.24
CXCL10	0.73 (0.55-0.96)	0.70 (0.52-0.95)	0.21
MIC A/B	0.71 (0.53-0.95)	0.70 (0.52-0.95)	0.22
SEZ6L	0.70 (0.50-1.00)	0.66 (0.46-0.96)	0.25
S100A4	0.70 (0.52-0.94)	0.66 (0.48-0.91)	0.13
FGF-23	0.69 (0.49-0.98)	0.65 (0.44-0.95)	0.24
TRAIL	0.70 (0.52-0.95)	0.62 (0.44-0.90)	0.13
HGF	0.65 (0.47-0.90)	0.60 (0.41-0.87)	0.11
ESM1	0.62 (0.46-0.85)	0.60 (0.43-0.84)	0.08

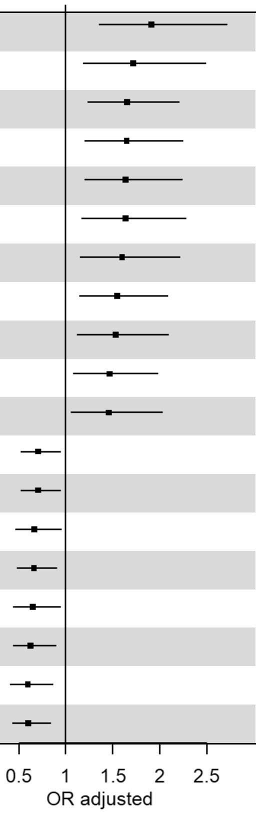

Table S5. Subgroup analyses

Colon	OR (95% CI) Crude	OR (95% CI) Adjusted*	P-value*
FGF21	1.25 (1.06-1.48)	1.23 (1.03-1.47)	0.02
MSLN	1.18 (0.99-1.41)	1.20 (0.98-1.45)	0.07
CEA	1.19 (0.99-1.42)	1.14 (0.94-1.38)	0.18
S100A11	1.16 (0.97-1.40)	1.11 (0.92-1.35)	0.27
hK8	1.09 (0.92-1.30)	1.10 (0.92-1.32)	0.28
IL8	1.10 (0.93-1.30)	1.10 (0.93-1.31)	0.27
CXCL10	1.03 (0.87-1.23)	1.05 (0.87-1.26)	0.62
IL6	1.06 (0.88-1.27)	1.00 (0.83-1.22)	0.98
HGF	1.09 (0.92-1.29)	1.00 (0.93-1.20)	0.98
LIF-R	0.99 (0.83-1.18)	1.00 (0.83-1.20)	0.98
IL-6RA	0.98 (0.82-1.17)	0.98 (0.82-1.18)	0.84
ENG	0.98 (0.82-1.17)	0.98 (0.81-1.18)	0.83
vWF	0.97 (0.79-1.18)	0.97 (0.79-1.19)	0.79
MIC-AB	0.96 (0.81-1.12)	0.96 (0.82-1.14)	0.67
ESM-1	0.93 (0.78-1.11)	0.96 (0.70-1.16)	0.70
EGFR	0.97 (0.80-1.16)	0.96 (0.89-1.16)	0.67
PPY	0.98 (0.82-1.18)	0.96 (0.80-1.16)	0.66
VEGFR-2	0.99 (0.83-1.18)	0.95 (0.89-1.13)	0.56
Dkk-1	0.94 (0.79-1.10)	0.92 (0.78-1.09)	0.35
TRAIL	0.95 (0.80-1.12)	0.88 (0.84-1.05)	0.15
5'-NT	0.86 (0.72-1.02)	0.83 (0.79-0.99)	0.04
**Rectum**	**OR (95% CI) Crude**	**OR (95% CI) Adjusted***	**P-value***
PPY	1.43 (1.10-1.86)	1.47 (1.12-1.92)	0.01
vWF	1.11 (0.86-1.44)	1.12 (0.85-1.47)	0.42
HGF	1.11 (0.89-1.39)	1.11 (0.86-1.43)	0.44
ESM-1	1.04 (0.83-1.30)	1.10 (0.86-1.41)	0.44
TRAIL	1.12 (0.88-1.41)	1.10 (0.86-1.41)	0.44
LIF-R	1.13 (0.88-1.45)	1.08 (0.83-1.39)	0.57
IL6	1.06 (0.84-1.33)	1.07 (0.84-1.37)	0.58
CXCL10	1.09 (0.87-1.37)	1.07 (0.85-1.35)	0.57
VEGFR-2	1.12 (0.88-1.41)	1.07 (0.83-1.36)	0.62
CEA	1.07 (0.85-1.35)	1.04 (0.80-1.35)	0.78
S100A11	1.02 (0.80-1.29)	1.04 (0.80-1.35)	0.78
FGF21	1.03 (0.83-1.29)	1.03 (0.82-1.29)	0.82
5'-NT	1.05 (0.84-1.32)	1.02 (0.80-1.29)	0.89
IL8	1.03 (0.82-1.28)	1.01 (0.80-1.27)	0.94
MIC-AB	1.02 (0.82-1.27)	1.01 (0.80-1.27)	0.95

ENG	0.98 (0.77-1.26)	0.98 (0.75-1.27)	0.87
EGFR	0.93 (0.73-1.20)	0.96 (0.74-1.24)	0.73
Dkk-1	0.92 (0.74-1.16)	0.95 (0.75-1.20)	0.66
MSLN	0.90 (0.72-1.14)	0.94 (0.72-1.23)	0.67
hK8	0.89 (0.70-1.13)	0.90 (0.70-1.16)	0.43
IL-6RA	0.84 (0.66-1.07)	0.87 (0.68-1.13)	0.30
**Stage I-II**	**OR (95% CI) Crude**	**OR (95% CI) Adjusted***	**P-value***
FGF21	1.37 (1.12-1.66)	1.35 (1.10-1.65)	0.00
CXCL10	1.09 (0.89-1.33)	1.09 (0.88-1.36)	0.41
IL6	1.18 (0.96-1.44)	1.09 (0.87-1.36)	0.47
MSLN	1.14 (0.93-1.40)	1.08 (0.86-1.37)	0.51
S100A11	1.15 (0.94-1.41)	1.07 (0.87-1.34)	0.51
CEA	1.15 (0.94-1.41)	1.06 (0.84-1.32)	0.64
IL8	1.08 (0.89-1.32)	1.05 (0.86-1.29)	0.63
HGF	1.12 (0.93-1.36)	1.03 (0.84-1.27)	0.75
ESM-1	0.95 (0.79-1.16)	1.02 (0.83-1.27)	0.83
PPY	1.05 (0.85-1.30)	1.01 (0.81-1.25)	0.96
ENG	1.03 (0.83-1.26)	1.00 (0.80-1.26)	1.00
vWF	1.04 (0.84-1.28)	1.00 (0.80-1.25)	0.97
hK8	0.97 (0.79-1.20)	0.99 (0.80-1.23)	0.96
VEGFR-2	1.04 (0.86-1.26)	0.99 (0.81-1.22)	0.95
LIF-R	0.94 (0.76-1.16)	0.94 (0.75-1.17)	0.55
MIC-AB	0.97 (0.80-1.17)	0.91 (0.75-1.12)	0.38
EGFR	0.90 (0.72-1.12)	0.91 (0.73-1.13)	0.39
5’-NT	0.92 (0.76-1.13)	0.88 (0.72-1.08)	0.23
IL-6RA	0.89 (0.73-1.10)	0.88 (0.71-1.09)	0.23
Dkk-1	0.88 (0.72-1.07)	0.88 (0.72-1.08)	0.22
TRAIL	0.96 (0.80-1.16)	0.87 (0.70-1.07)	0.18
**Stage III-IV**	**OR (95% CI) Crude**	**OR (95% CI) Adjusted***	**P-value***
CEA	1.15 (0.94-1.40)	1.18 (0.95-1.48)	0.14
LIF-R	1.17 (0.95-1.44)	1.17 (0.95-1.44)	0.15
PPY	1.11 (0.91-1.36)	1.10 (0.89-1.35)	0.38
MSLN	1.02 (0.84-1.25)	1.07 (0.86-1.33)	0.54
S100A11	1.07 (0.86-1.34)	1.06 (0.85-1.33)	0.59
hK8	1.03 (0.84-1.26)	1.05 (0.85-1.30)	0.63
VEGFR-2	1.06 (0.86-1.31)	1.05 (0.85-1.30)	0.65
IL8	1.02 (0.84-1.23)	1.02(0.85-1.24)	0.81
EGFR	1.03 (0.84-1.28)	1.02 (0.82-1.27)	0.85
ESM-1	0.99 (0.81-1.21)	1.02 (0.82-1.26)	0.87
HGF	1.04 (0.86-1.26)	1.01 (0.82-1.25)	0.93
ENG	0.96 (0.78-1.18)	0.99 (0.80-1.23)	0.95
IL-6RA	0.99 (0.80-1.22)	0.99 (0.79-1.23)	0.91

Dkk-1	0.98 (0.82-1.19)	0.99 (0.82-1.19)	0.89
FGF21	1.02 (0.85-1.24)	0.98 (0.80-1.21)	0.88
CXCL10	1.02 (0.84-1.23)	0.98 (0.80-1.20)	0.86
MIC-AB	0.98 (0.81-1.18)	0.97 (0.80-1.18)	0.77
vWF	0.98 (0.76-1.25)	0.97 (0.76-1.24)	0.80
TRAIL	0.98 (0.80-1.21)	0.96 (0.77-1.18)	0.67
IL6	0.94 (0.77-1.15)	0.90 (0.73-1.12)	0.35
5'-NT	0.91 (0.75-1.11)	0.88 (0.72-1.08)	0.22
>5 years until diagnosis	OR (95% CI) Crude	OR (95% CI) Adjusted*	P-value*
MSLN	1.15 (0.98-1.34)	1.20 (1.01-1.43)	0.04
FGF21	1.23 (1.06-1.42)	1.19 (1.02-1.38)	0.02
PPY	1.17 (0.99-1.37)	1.15 (0.97-1.36)	0.10
CEA	1.15 (0.99-1.34)	1.13 (0.96-1.34)	0.15
vWF	1.11 (0.93-1.33)	1.10 (0.92-1.32)	0.29
S100A11	1.12 (0.95-1.31)	1.07 (0.91-1.27)	0.41
LIF-R	1.03 (0.88-1.21)	1.03 (0.88-1.21)	0.70
HGF	1.09 (0.94-1.27)	1.03 (0.87-1.21)	0.74
CXCL10	1.04 (0.90-1.21)	1.02 (0.88-1.20)	0.76
VEGFR-2	1.06 (0.91-1.24)	1.02 (0.87-1.20)	0.78
hK8	1.00 (0.85-1.17)	1.02 (0.87-1.20)	0.79
IL6	1.08 (0.92-1.26)	1.01 (0.86-1.19)	0.89
MIC-AB	1.01 (0.88-1.17)	1.01 (0.87-1.16)	0.94
TRAIL	1.04 (0.89-1.20)	0.99 (0.84-1.15)	0.86
IL8	0.98 (0.85-1.14)	0.98 (0.85-1.14)	0.81
IL-6RA	0.99 (0.84-1.16)	0.98 (0.83-1.15)	0.78
ENG	0.94 (0.81-1.10)	0.97 (0.82-1.14)	0.73
ESM-1	0.90 (0.78-1.05)	0.96 (0.81-1.13)	0.60
EGFR	0.95 (0.80-1.11)	0.94 (0.80-1.12)	0.50
5'-NT	0.97 (0.84-1.12)	0.93 (0.80-1.08)	0.33
Dkk-1	0.92 (0.79-1.06)	0.93 (0.80-1.07)	0.30
<=5 years until diagnosis	OR (95% CI) Crude	OR (95% CI) Adjusted*	P-value*
IL8	1.57 (1.07-2.31)	1.55 (1.03-2.33)	0.04
ESM-1	1.35 (0.98-1.87)	1.39 (1.03-1.98)	0.07
ENG	1.30 (0.92-1.83)	1.24 (0.86-1.80)	0.25
CXCL10	1.16 (0.83-1.61)	1.18 (0.83-1.67)	0.35
S100A11	1.15 (0.82-1.62)	1.17 (0.81-1.68)	0.40
HGF	1.15 (0.86-1.55)	1.12 (0.81-1.54)	0.51
hK8	1.10 (0.82-1.47)	1.10 (0.80-1.50)	0.56
EGFR	1.10 (0.79-1.52)	1.08 (0.76-1.54)	0.66
Dkk-1	1.08 (0.78-1.49)	1.06 (0.75-1.49)	0.73
CEA	1.11 (0.81-1.54)	1.03 (0.68-1.56)	0.89

VEGFR-2	1.01 (0.76-1.35)	1.01 (0.73-1.40)	0.94
LIF-R	1.07 (0.77-1.48)	1.00 (0.70-1.43)	1.00
IL6	0.95 (0.68-1.31)	0.98 (0.69-1.39)	0.90
FGF21	0.92 (0.68-1.24)	0.91 (0.65-1.26)	0.56
IL-6RA	0.81 (0.57-1.15)	0.85 (0.59-1.24)	0.40
TRAIL	0.85 (0.61-1.16)	0.85 (0.60-1.20)	0.34
MIC-AB	0.88 (0.65-1.17)	0.84 (0.62-1.16)	0.29
PPY	0.90 (0.65-1.25)	0.81 (0.57-1.16)	0.26
vWF	0.76 (0.53-1.07)	0.72 (0.49-1.05)	0.09
5'-NT	0.79 (0.56-1.10)	0.71 (0.49-1.04)	0.08
MSLN	0.83 (0.61-1.13)	0.71 (0.48-1.03)	0.07
KRAS mut.	OR (95% CI) Crude	OR (95% CI) Adjusted*	P-value*
PPY	1.38 (0.96-1.99)	1.34 (0.89-2.00)	0.16
IL8	1.33 (0.97-1.81)	1.30 (0.93-1.82)	0.12
FGF21	1.28 (0.95-1.71)	1.19 (0.87-1.63)	0.27
vWF	1.19 (0.82-1.72)	1.17 (0.79-1.73)	0.44
TRAIL	1.20 (0.91-1.60)	1.16 (0.86-1.55)	0.34
CXCL10	1.15 (0.85-1.56)	1.14 (0.82-1.58)	0.43
ESM-1	1.05 (0.80-1.38)	1.14 (0.84-1.54)	0.41
IL-6RA	1.18 (0.85-1.63)	1.13 (0.81-1.59)	0.47
HGF	1.26 (0.92-1.73)	1.11 (0.78-1.60)	0.56
LIF-R	1.17 (0.86-1.59)	1.09 (0.78-1.53)	0.60
CEA	1.21 (0.91-1.62)	1.08 (0.76-1.54)	0.65
IL6	1.21 (0.89-1.65)	1.08 (0.76-1.52)	0.67
ENG	1.01 (0.75-1.36)	1.07 (0.77-1.48)	0.71
Dkk-1	1.06 (0.79-1.41)	1.03 (0.76-1.41)	0.83
MSLN	1.00 (0.74-1.35)	1.02 (0.68-1.53)	0.92
S100A11	1.06 (0.78-1.45)	1.01 (0.73-1.40)	0.96
hK8	0.99 (0.71-1.38)	1.00 (0.69-1.43)	0.98
5'-NT	1.04 (0.78-1.39)	0.99 (0.73-1.35)	0.96
VEGFR-2	1.11 (0.81-1.53)	0.96 (0.67-1.36)	0.81
EGFR	1.03 (0.75-1.41)	0.95 (0.68-1.33)	0.77
MIC-AB	0.68 (0.50-0.93)	0.66 (0.47-0.93)	0.02
BRAF mut.	OR (95% CI) Crude	OR (95% CI) Adjusted*	P-value*
CEA	1.35 (0.93-1.95)	1.47 (0.93-2.33)	0.10
S100A11	1.39 (0.98-1.99)	1.34 (0.89-2.01)	0.16
HGF	1.34 (0.95-1.89)	1.29 (0.88-1.88)	0.19
MSLN	1.19 (0.84-1.67)	1.26 (0.84-1.89)	0.27
CXCL10	1.26 (0.90-1.75)	1.21 (0.85-1.73)	0.29
MIC-AB	1.20 (0.88-1.64)	1.17 (0.85-1.63)	0.32
FGF21	1.22 (0.88-1.68)	1.17 (0.82-1.65)	0.39

ESM-1	0.99 (0.68-1.44)	1.16 (0.76-1.78)	0.49
VEGFR-2	1.18 (0.82-1.69)	1.14 (0.78-1.66)	0.49
PPY	1.05 (0.77-1.43)	1.10 (0.78-1.54)	0.59
Dkk-1	1.06 (0.76-1.47)	1.05 (0.74-1.48)	0.80
hK8	0.98 (0.72-1.34)	1.04 (0.75-1.44)	0.81
IL8	1.01 (0.73-1.39)	0.99 (0.70-1.40)	0.95
ENG	0.95 (0.67-1.36)	0.97 (0.65-1.45)	0.89
IL6	1.08 (0.74-1.56)	0.96 (0.63-1.45)	0.85
LIF-R	0.91 (0.64-1.30)	0.95 (0.66-1.38)	0.79
vWF	0.90 (0.62-1.30)	0.84 (0.57-1.25)	0.39
TRAIL	0.95 (0.67-1.36)	0.84 (0.57-1.25)	0.39
IL-6RA	0.85 (0.56-1.29)	0.79 (0.51-1.23)	0.30
EGFR	0.83 (0.53-1.29)	0.77 (0.48-1.25)	0.29
5'-NT	0.82 (0.57-1.17)	0.77 (0.52-1.14)	0.20

BRAF and *KRAS* wild type	OR (95% CI) Crude	OR (95% CI) Adjusted*	P-value*
FGF21	1.12 (0.95-1.32)	1.10 (0.93-1.30)	0.28
MSLN	1.08 (0.91-1.28)	1.08 (0.90-1.30)	0.42
MIC-AB	1.08 (0.91-1.28)	1.07 (0.90-1.27)	0.44
PPY	1.08 (0.90-1.30)	1.07 (0.88-1.28)	0.51
hK8	1.04 (0.87-1.24)	1.05 (0.87-1.26)	0.61
LIF-R	1.04 (0.86-1.25)	1.05 (0.87-1.26)	0.64
CEA	1.07 (0.90-1.28)	1.04 (0.86-1.25)	0.72
vWF	1.03 (0.85-1.26)	1.03 (0.84-1.26)	0.76
ENG	1.02 (0.85-1.22)	1.02 (0.85-1.24)	0.80
S100A11	1.03 (0.86-1.24)	1.01 (0.83-1.23)	0.91
IL8	1.01 (0.86-1.20)	1.01 (0.85-1.20)	0.90
EGFR	0.99 (0.83-1.18)	1.01 (0.84-1.21)	0.92
VEGFR-2	1.03 (0.87-1.21)	1.00 (0.84-1.19)	1.00
CXCL10	0.99 (0.83-1.18)	0.99 (0.83-1.19)	0.94
ESM-1	0.95 (0.80-1.13)	0.99 (0.82-1.19)	0.89
HGF	1.02 (0.87-1.20)	0.98 (0.82-1.18)	0.85
IL6	1.01 (0.85-1.20)	0.98 (0.82-1.17)	0.83
IL-6RA	0.92 (0.77-1.10)	0.93 (0.78-1.11)	0.44
5'-NT	0.94 (0.79-1.11)	0.91 (0.77-1.08)	0.29
TRAIL	0.94 (0.79-1.12)	0.90 (0.75-1.08)	0.26
Dkk-1	0.88 (0.74-1.04)	0.89 (0.75-1.05)	0.17

*Adjusted for BMI, smoking and education and conditioned on matching criteria (age, sex, sampling date, fasting status and number of freeze-thaw cycles).

Table S6. Proteins selected in the Lasso regression models.

Model	Selected proteins	AUC (95% CI) Baseline	AUC (95% CI) Lasso model	p-value (log-likelihood)
**Full cohort**	FGF-21	0.55 (0.51-0.59)	0.57 (0.53-0.60)	<0.05
**Colon**	5'NT, MSLN, Dkk1, FGF-21, S100A11, CEA	0.56 (0.52-0.61)	0.63 (0.59-0.68)	<0.01
**Rectum**	PPY	0.57 (0.51-0.64)	0.61 (0.55-0.67)	<0.01
**Stage I-II**	FGF-21	0.58 (0.53-0.64)	0.62 (0.57-0.67)	<0.01
**Stage III-IV**	PPY, IL6, LIF-R, 5'NT, ENG, CEA	0.55 (0.50-0.61)	0.60 (0.54-0.65)	0.25
**>5 years until diagnosis**	PPY, MSLN, Dkk1, FGF21, CEA	0.55 (0.51-0.59)	0.60 (0.56-0.64)	0.08
**<=5 years until diagnosis**	All 21	0.59 (0.51-0.67)	0.73 (0.65-0.80)	0.29
***KRAS* mut.**	PPY, IL8, MIC-AB, TRAIL, FGF21, CEA	0.59 (0.51-0.67)	0.67 (0.60-0.75)	0.06
***BRAF* mut.**	All 21	0.58 (0.49-0.67)	0.70 (0.62-0.79)	0.72
***KRAS* and *BRAF* wild type**	All 21	0.55 (0.50-0.60)	0.59 (0.54-0.64)	0.98

References

1 Myte, R., Sundkvist, A., Guelpen, B. & Harlid, S. Circulating levels of inflammatory markers and DNA methylation, an analysis of repeated samples from a population based cohort. *Epigenetics* **14**, 649-659, doi:10.1080/15592294.2019.1603962 (2019).

2 Rho, J.-h. *et al.* Protein and glycomic plasma markers for early detection of adenoma and colon cancer. *Gut* **67**, 473, doi:10.1136/gutjnl-2016-312794 (2018).

3 Cohen, J. D. *et al.* Detection and localization of surgically resectable cancers with a multi-analyte blood test. *Science* **359**, 926-930, doi:10.1126/science.aar3247 (2018).

4 Qi, L. *et al.* Dickkopf-1 inhibits epithelial-mesenchymal transition of colon cancer cells and contributes to colon cancer suppression. *Cancer Science* **103**, 828-835, doi:10.1111/j.1349-7006.2012.02222.x (2012).

5 Li, S. *et al.* Plasma Mesothelin as a Novel Diagnostic and Prognostic Biomarker in Colorectal Cancer. *Journal of Cancer* **8**, 1355-1361, doi:10.7150/jca.18014 (2017).
